# Microcrystals in structural biology: small samples, big insights

**DOI:** 10.1107/S2052252525003653

**Published:** 2025-04-28

**Authors:** Dominik Oberthür

**Affiliations:** a Center for Free-Electron Laser Science CFEL Deutsches Elektronen-Synchrotron DESY Notkestr. 85 22607Hamburg Germany

**Keywords:** structural biology, macromolecular crystallography, microcrystals, time-resolved studies, MicroED, serial crystallography

## Abstract

Microcrystals are transforming structural biology by enabling high-resolution structures and time-resolved insights from samples once deemed too small. This commentary highlights recent advances in microfocus X-ray and MicroED methods, emphasizing their growing role as powerful and complementary tools in modern macromolecular crystallography.

Structural biologists have long sought ever larger and more perfect crystals of proteins and other biological macromolecules to determine their structures through crystallography. In the early days of X-ray crystallography, protein crystals often needed to be at least tenths of a millimetre in size to yield usable diffraction data. Today, however, a significant shift is underway: even microcrystals – just a few micrometres in size or smaller – are now delivering high-resolution structures.

In the current issue of *IUCrJ*, Tremlett *et al.* offer a sweeping overview of how these ‘small but mighty’ crystals are reshaping structural biology (Tremlett *et al.*, 2025 ▸). One of the key contributions of their review is its contextualization of microcrystals not as a niche solution, but as a central strategy. What was once viewed as a limitation – only obtaining tiny crystals – has been re-imagined as an opportunity, thanks to cutting-edge beamlines like VMXm at the Diamond Light Source and cryogenic electron microscopes adapted for MicroED (see Fig. 1 ▸). The authors trace this evolution from pioneering microfocus beamlines at the ESRF to the ultra-high-resolution structures now achievable via MicroED (Martynowycz *et al.*, 2022 ▸). These developments are not merely salvage pathways for intractable projects – they open entirely new avenues for structural insight. In short, MicroED and advanced microfocus X-ray setups now offer complementary routes for structure determination from crystals previously considered too small (Tremlett *et al.*, 2025 ▸).

Beyond making microcrystals viable, advances in X-ray sources – such as X-ray free-electron lasers (XFELs) and fourth-generation synchrotrons – have fundamentally changed how data are collected. Serial crystallography (Henkel & Oberthür, 2024 ▸) is a paradigm-shifting approach in which diffraction data are collected from a stream or series of thousands of microcrystals, rather than a single large one. First pioneered at the XFEL Linac Coherent Light Source (LCLS) in 2009 (Chapman *et al.*, 2011 ▸), this technique revolutionized data collection through the use of ultra-short X-ray pulses and fresh crystals for each exposure – effectively outrunning radiation damage. It also enabled routine structure determination at room temperature, allowing proteins to be studied in more physiologically relevant states (Henkel & Oberthür, 2024 ▸).

One of the most exciting applications of serial crystallography is time-resolved structural biology – essentially making molecular stop-motion movies (Caramello & Royant, 2024 ▸). By triggering a reaction in microcrystals and collecting diffraction snapshots at various time points, researchers can capture structural changes as they occur. Techniques such as mix-and-inject serial crystallography (MISC) and pump–probe experiments allow reaction intermediates to be captured with temporal resolutions ranging from milliseconds to femtoseconds. Thanks to properties intrinsic to microcrystals – namely, minimal diffusion barriers for rapid substrate access and thicknesses tuned to fall within the absorption depth of the excitation light – these methods enable near-instantaneous substrate entry and uniform photoactivation, both essential for studying enzyme catalysis and allosteric regulation. However, activation via substrate mixing is inherently limited to timescales larger than microseconds (Schmidt, 2013 ▸), restricting its applicability for capturing ultra-fast processes.

Harnessing the potential of microcrystals requires innovation in sample preparation and delivery. Tremlett *et al.* dedicate much attention to the practical challenge of reliably producing high-quality microcrystals, reviewing the literature and offering their own perspectives. They provide practical guidance on transforming macrocrystals into microcrystal slurries, either top-down via crushing or filtering, or bottom-up through seeding. They also discuss the use of phase diagrams to understand crystallization behaviour, scaling up from tiny vapour diffusion droplets to batch preparations, and novel microfluidic crystallization techniques. Importantly, they emphasize that no universal recipe exists; success lies in tailoring the preparation method to the experimental modality.

Once microcrystals are prepared, delivering them efficiently to the X-ray or electron beam is the next hurdle. Their review summarizes a diverse array of delivery techniques developed for MicroED and serial crystallography. Each approach comes with trade-offs in terms of sample usage, speed and technical complexity. The choice of delivery method depends on the experimental priorities, be it rapid mixing, low background or minimal sample consumption. Encouragingly, a growing toolbox of delivery strategies now empowers researchers to tackle projects that once seemed unfeasible due to sample limitations.

The embrace of microcrystals marks a profound shift in structural biology. Previously, success often depended on painstakingly growing large, well ordered crystals (Chayen & Saridakis, 2008 ▸) and optimizing conditions for cryo-cooling. Today, the synergy of modern techniques reviewed by Tremlett *et al.* allows scientists to follow a different path: grow many microcrystals and apply advanced microcrystallography to answer structural questions. These methods not only rescue projects that yield only tiny crystals, but also enable wholly new experiments – especially time-resolved studies of macromolecular dynamics – that were out of reach with traditional crystallography.

It is important to note that the rise of microcrystal-based methods complements the revolution in single-particle cryo-electron microscopy (cryo-EM), which solves structures without any crystals at all. Each technique has its niche: MicroED excels at atomic resolution structures of small proteins or polymorphs, while serial femtosecond crystallography captures ultra-fast motions in enzymes or during signal transduction. Cryo-EM, by contrast, is ideal for large complexes and snapshots of flexible assemblies, while cryo-electron tomography (cryo-ET) can capture supermolecular complexes in their native environment, albeit at reduced spatial resolution (Berger *et al.*, 2023 ▸). Together, these advances provide structural biologists with an unprecedented toolkit.

As Tremlett *et al.* conclude, microcrystallography techniques ‘hold great promise for tackling some of the most challenging questions in structural biology’. Realizing that promise will depend on choosing the right technique for each biological problem. Continued refinement of crystallization methods, such as new microfluidic approaches (Pachl *et al.*, 2025 ▸) to precisely control crystal size (Stubbs *et al.*, 2024 ▸), will further reduce the difficulty of preparing microcrystals. In turning these tiny crystals into powerful structural tools, the field of structural biology is now poised to explore proteins and their dynamics with a clarity and scope once thought impossible. The little crystals that could are now driving big discoveries.

## Figures and Tables

**Figure 1:**
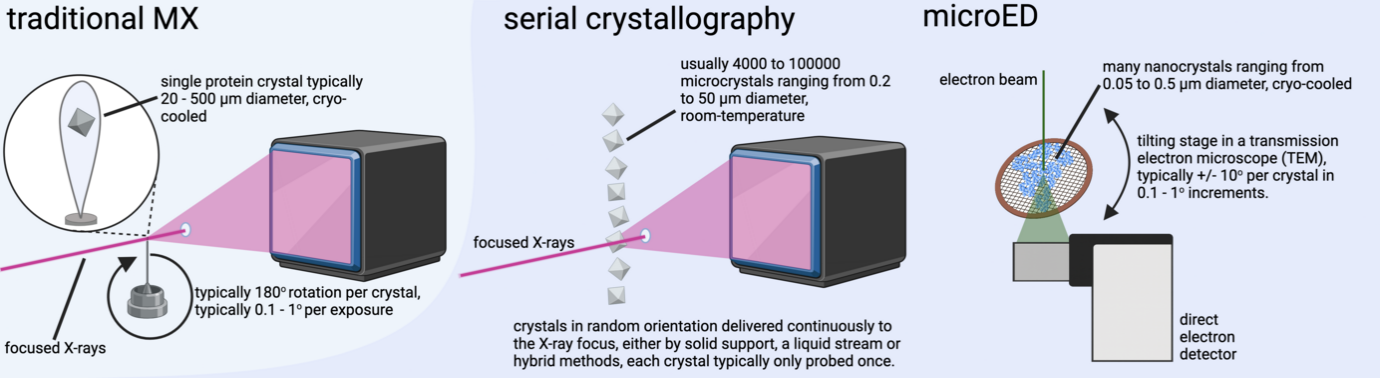
Microcrystals powering modern structure determination: from X-rays to electrons. Traditional macromolecular crystallography (MX) compared with serial crystallography at X-ray free-electron lasers (XFELs) and synchrotrons, and MicroED using a transmission electron microscope. Traditional MX relies on single, cryo-cooled crystals of ~20–500 µm diameter, typically rotated through ~180° with exposures of 0.1–1°. In contrast, serial crystallography uses thousands of microcrystals (typically 0.2–50 µm), delivered at room temperature in random orientations, with each crystal exposed only once to a focused X-ray pulse. MicroED involves electron diffraction from cryo-cooled nanocrystals (typically 0.05–0.5 µm) mounted on a tilted EM grid, typically collecting ±10° of data per crystal. (Image created with *BioRender*, https://www.biorender.com.)

## References

[bb1] Berger, C., Premaraj, N., Ravelli, R. B. G., Knoops, K., López-Iglesias, C. & Peters, P. J. (2023). *Nat. Methods*, **20**, 499–511. 10.1038/s41592-023-01783-5 36914814

[bb2] Caramello, N. & Royant, A. (2024). *Acta Cryst.* D**80**, 60–79. 10.1107/S2059798323011002 PMC10836399 38265875

[bb3] Chapman, H. N., Fromme, P., Barty, A., White, T. A., Kirian, R. A., Aquila, A., Hunter, M. S., Schulz, J., DePonte, D. P., Weierstall, U., Doak, R. B., Maia, F. R. N. C., Martin, A. V., Schlichting, I., Lomb, L., Coppola, N., Shoeman, R. L., Epp, S. W., Hartmann, R., Rolles, D., Rudenko, A., Foucar, L., Kimmel, N., Weidenspointner, G., Holl, P., Liang, M., Barthelmess, M., Caleman, C., Boutet, S., Bogan, M. J., Krzywinski, J., Bostedt, C., Bajt, S., Gumprecht, L., Rudek, B., Erk, B., Schmidt, C., Hömke, A., Reich, C., Pietschner, D., Strüder, L., Hauser, G., Gorke, H., Ullrich, J., Herrmann, S., Schaller, G., Schopper, F., Soltau, H., Kühnel, K.-U., Messerschmidt, M., Bozek, J. D., Hau-Riege, S. P., Frank, M., Hampton, C. Y., Sierra, R. G., Starodub, D., Williams, G. J., Hajdu, J., Timneanu, N., Seibert, M. M., Andreasson, J., Rocker, A., Jönsson, O., Svenda, M., Stern, S., Nass, K., Andritschke, R., Schröter, C.-D., Krasniqi, F., Bott, M., Schmidt, K. E., Wang, X., Grotjohann, I., Holton, J. M., Barends, T. R. M., Neutze, R., Marchesini, S., Fromme, R., Schorb, S., Rupp, D., Adolph, M., Gorkhover, T., Andersson, I., Hirsemann, H., Potdevin, G., Graafsma, H., Nilsson, B. & Spence, J. C. H. (2011). *Nature*, **470**, 73–77.

[bb4] Chayen, N. E. & Saridakis, E. (2008). *Nat. Methods*, **5**, 147–153. 10.1038/nmeth.f.203 18235435

[bb5] Henkel, A. & Oberthür, D. (2024). *Acta Cryst.* D**80**, 563–579. 10.1107/S2059798324005588 PMC11301758 38984902

[bb6] Martynowycz, M. W., Clabbers, M. T. B., Hattne, J. & Gonen, T. (2022). *Nat. Methods*, **19**, 724–729. 10.1038/s41592-022-01485-4 PMC9184278 35637302

[bb7] Pachl, P., Coudray, L., Vincent, R., Nilles, L., Scheer, H., Ritzenthaler, C., Fejfarová, A., Řezáčová, P., Engilberge, S. & Sauter, C. (2025). *FEBS Open Bio*, **15**, 532–541. 10.1002/2211-5463.13932 PMC11961392 39572886

[bb20] Schmidt, M. (2013). *Adv. Condens. Matter Phys.* **2013**, e167276.

[bb8] Stubbs, J., Hornsey, T., Hanrahan, N., Esteban, L. B., Bolton, R., Malý, M., Basu, S., Orlans, J., de Sanctis, D., Shim, J. U., Shaw Stewart, P. D., Orville, A. M., Tews, I. & West, J. (2024). *IUCrJ*, **11**, 237–248. 10.1107/S2052252524001799 PMC10916287 38446456

[bb9] Tremlett, C. J., Stubbs, J., Stuart, W. S., Shaw Stewart, P. D., West, J., Orville, A. M., Tews, I. & Harmer, N. J. (2025). *IUCrJ*, **12**, 262–279. 10.1107/S2052252525001484 PMC12044856 40080159

